# Individualized Goal Directed Perioperative Care – The Way to Go!

**DOI:** 10.3389/fmed.2015.00022

**Published:** 2015-04-08

**Authors:** Zsolt Molnár

**Affiliations:** ^1^Department of Anaesthesiology and Intensive Therapy, Faculty of Medicine, University of Szeged, Szeged, Hungary

**Keywords:** high-risk surgery, goal directed therapy, pulse pressure variation, central venous oxygen saturation, stroke volume, improved outcome

Treating critically ill and high-risk patients is one of the greatest challenges in medicine, and it is also a multidisciplinary task. Practice varies country by country resulting in a large scatter in outcome data, which makes the interpretation difficult. Regarding anesthesia and surgery, based on an international 7 day cohort, the EUSOS-study, conducted in 28 countries in Europe including 46,000 patients undergoing inpatient surgery, the overall mortality was 4%, which was higher than expected (1). What is even more surprising that 73% of the patients who died were never admitted to an intensive care unit (ICU).

One of the leading reasons of an unfavorable outcome is inadequate perioperative hemodynamic management. Although this statement is widely accepted, yet fluids and catecholamines are commonly prescribed to subjective criteria (2).

Several large prospective randomized trials have recently studied the effects of advanced hemodynamic monitoring based perioperative goal-directed therapy (3–7). There is also gathering evidence in other fields of perioperative care that patients should be treated according to individualized, physiology based values rather than guidelines or treatment bundles determined values (8).

These results may lead to a paradigm shift in critical care medicine and change our practice from a protocolized “figure based” management to treating patients according to their individual and actual needs.

## Fluid Therapy and Outcome

The main task of any type of resuscitation is to maintain adequate balance between oxygen delivery (DO_2_) and consumption (VO_2_), which is normally around 25–30% (VO_2_/DO_2_) in an awake adult at rest. Fluid therapy has a pivotal role in maintaining hemodynamic stability, and it is important to acknowledge that the only reason patients need infusion is to normalize DO_2_ by increasing stroke volume hence cardiac output (CO). Several studies revealed that both intraoperative hypo- and hypervolemia can be responsible for post-operative complications and organ failure, while optimization of perioperative hemodynamics may result in improved outcome (3, 4, 9, 10). Rather than taking sides on “restrictive” or “liberal” fluid management, careful monitoring of the hemodynamic status may be more beneficial in tailoring fluid therapy for the patients’ actual needs.

According to a recent survey, most anesthetists rely on conventional measures during the management of high-risk patients (2). However, using heart rate, mean arterial pressure (MAP) and central venous pressure (CVP) only, to assess the hemodynamic status may be misleading (11, 12). This conventional approach may be adequate for most patients undergoing surgical procedures, but the relatively small number of patients at high risk of complications and even death may need advanced monitoring guided care. To optimize perioperative hemodynamic status of this high-risk population, the most frequently used parameters next to standard monitoring are CO and CO-driven variables, and/or DO_2_. CO can be measured by several ways. The use of intermittent thermodilution by the pulmonary artery catheter has been decreased in surgical patients over the past decades, although it may still have a role in medical patients (13). Less invasive ways are transpulmonary thermodilution or other non-calibrated, pulse contour analysis based technologies. Furthermore, using non-invasive echocardiography enables physicians to measure CO, intravascular pressures and volumes, systolic and diastolic function of both ventricles, and preload responsiveness. Although outcome studies are still warranted, but quick assessment of patients with echocardiography on the ICU or monitoring patients with esophageal Doppler in the operating theatre can be extremely useful and becoming more-and-more popular in the everyday routine (14).

In two recent animal experiments, we investigated CO and stroke volume guided hemorrhage and fluid resuscitation in a porcine model (15, 16). After baseline measurements, animals were bled until cardiac index (CI) or stroke volume index (SVI) dropped by 50%, after which animals were resuscitated over 60 min till baseline CI and SVI values were reached. In the CI-group, stroke volume, global end diastolic volume, and central venous oxygen saturation remained significantly lower, while stroke volume variation (SVV), central venous-to-arterial carbon dioxide difference (dCO_2_) remained significantly higher by the end of resuscitation as compared to baseline, indicating that fluid resuscitation might had been inadequate and the normalization of CI was mainly due to the persistently elevated heart rate, rather than restoration of the circulating blood volume. On the contrary in the SVI group, by the end of resuscitation, SVV, central venous oxygen saturation (ScvO_2_), and central venous–arterial carbon dioxide gap (dCO_2_) improved significantly or returned to their baseline value by the end of the experiment. Based on these experiments, it seems that the SVI-based algorithm resulted in better hemodynamic and oxygenation indices as compared to the CI-based approach; however, it should be tested in a randomized fashion.

**Figure 1:**
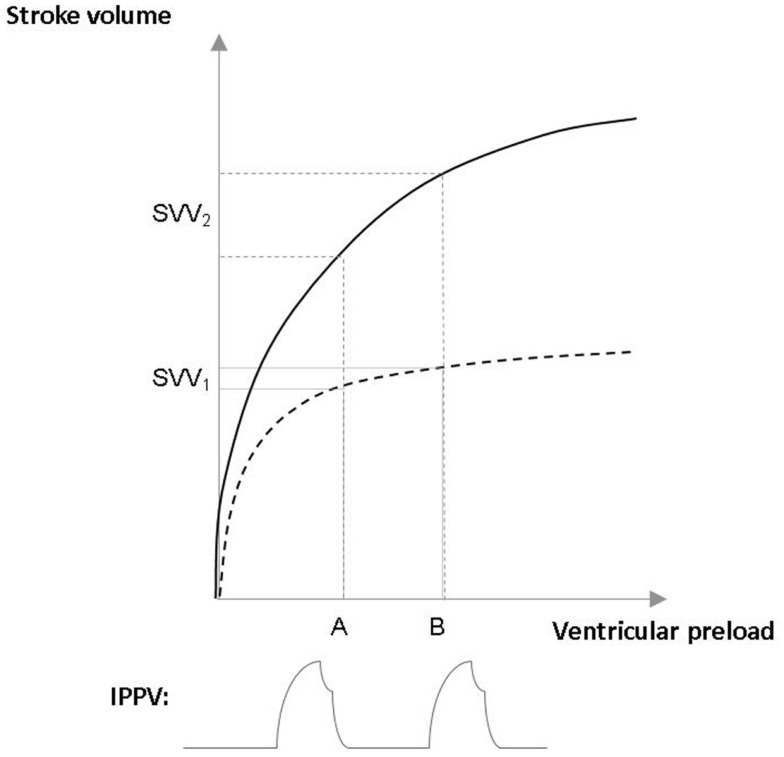
**Assessing fluid responsiveness with stroke volume variation (SVV) in patients with different Frank–Starling curves**. The upper curve (——) represents the Frank–Starling curve of a patient with normal heart, the lower curve (– – –) a patient with chronic heart failure. Intermittent positive pressure ventilation (IPPV) causes a similar changes in ventricular preload (A–B) in both cases. However, in the patient who is on the flat part on the Frank–Starling curve IPPV causes small stroke volume variation (SVV_1_), hence this patient would not respond for fluid administration with an increase in stroke volume. On the contrary, in the other case, the patient is on the steep part of the curve have large changes in stroke volume (SVV_2_), indicating fluid responsiveness.

According to the survey by Cannesson et al., dynamic indices such as SVV, pulse pressure variation (PPV) are only applied in 6–25% during anesthesia for high-risk surgery (2). Heart–lung interactions during forced expiration against closed glottis, which was first described by Antonio Mario Valsalva almost 300 years ago, form the principle of SVV/PPV phenomena. Intermittent positive pressure ventilation can be regarded as a series of Valsalva-maneuvers: large enough tidal volumes of ~8 mL/kg of ideal bodyweight (17), will cause decreased venous return, hence a drop in stroke volume, which can be detected by SVV/PPV. If the circulation is well filled the variation is small, while on the contrary, increased variation means hypovolemia (Figure 1). It is also important to acknowledge that patients have different types of Frank–Starling curves. This is why set figures of “static” preload indices, such as CVP, cannot indicate fluid responsiveness, also demonstrated in Figure 1 (18). Indeed, the variation in PPV induced by mechanical ventilation has been shown to be a very accurate predictor of fluid responsiveness, with an optimal threshold value around 12% (19). However, PPV/SVV based approaches have some limitations: it requires controlled ventilation with tidal volumes above the “protective” 6 mL/kg tidal volumes, and in the case of spontaneous breathing or breathing efforts and cardiac arrhythmias values cannot be relied on.

In a large multicenter randomized study, we recently reported that a PPV, MAP, and CI-trending based approach nearly halved post-operative complications in high-risk surgical patients undergoing major abdominal surgery (4). Detailed analysis revealed that patients undergoing bowel surgery benefited the most from this intervention, and the only difference between the treatment and control groups was that while no patients received positive inotropic agents in the control group, but 42% of patients required dobutamine in the treatment group.

The results of the OPTIMISE study were less convincing (5). Seven hundred thirty-four patients were randomized into control and CO-guided groups and they found no significant difference between the groups in the primary end-point of a composite outcome of complications and 30-day mortality. However, there was a measurable treatment effect, and at 180 days there was a non-significant reduction in mortality.

In a very recent clinical trial from the same group, goal directed DO_2_-therapy was applied in high-risk surgical patients needing ICU admission after the operation (7). Target DO_2_ was based on the preoperatively determined individual DO_2_ values. In the treatment group after the first hour of optimization 40% of patients received dobutamine as compared to none in controls, a very similar result also reported by us (4). Their main finding was that achievement of preoperative oxygen delivery soon after major surgery was associated with a reduction in early post-operative morbidity, yet this occurred irrespective of additional post-operative hemodynamic manipulation. This was in part due to the fact that more patients achieved the target DO_2_ in the control group (53%) than expected. Their data also suggest that dobutamine use resulted in the development of parasympathetic autonomic dysfunction, which can be harmful and should be investigated in the future.

In the POEMAS study, CO-measurement based algorithm was applied, using a non-invasive device (6). Goal directed therapy was managed according to arterial blood pressure, CI, and stroke volume response. Patients in this group received significantly more colloid boluses, red blood cell transfusion, and more patients required dobutamine (25%). They observed a significant reduction in reoperations in the treated group (5.6 vs. 15.7%). However, there were no significant differences in the overall complications.

It is important to acknowledge that invasive hemodynamic monitoring based care is not easy. It takes a lot of time, effort, and practice to put the data provided by these monitors into good use (13). Participants of the research groups may vary in experience, which can increase the noise-to-signal ratio in these trials, which may explain to some extent the diversity between the results of these studies. Nevertheless, there is a definitive signal, or at least a trend, which indicates that hemodynamic stabilization guided by CO and CO-driven indices could be beneficial in high-risk patients.

## Assessing and Treating Oxygen Debt

Although global macro-hemodynamic indices are very important but there is more to the picture. Another part of this “hemodynamic puzzle” not to be overlooked is the assessment of the VO_2_/DO_2_ balance. The most often used parameters to estimate the relationship between oxygen supply and consumption are mixed venous saturation (SvO_2_) and ScvO_2_. Although SvO_2_ is regarded as the most accurate indicator of the balance between global VO_2_/DO_2_, there is very good evidence that ScvO_2_ may serve as an easily obtainable and reliable alternative to manage therapy in critical ill patients (20). Continuous monitoring of the ScvO_2_ is also possible with a device based on fiberoptic technology and the measurements showed good correlation with laboratory values (21).

It has been shown that following major surgery reductions in ScvO_2_ are independently associated with post-operative complications (22). In another study, keeping oxygen extraction ratio below 27% resulted in less post-operative organ dysfunction and reduced hospital stay in high-risk surgical patients (23). There is limited data about the role of ScvO_2_-guided intra-, and perioperative management, but it is certainly something to be investigated in the future.

Hemoglobin is also one of the main determinants of DO_2_. In a number of guidelines, the indication of blood transfusion is based on certain levels of hemoglobin, usually 70–100 g/L (24). Higher values are recommended in patients with ischemic heart disease, and lower values for patients without the history of cardiac dysfunction. However, “ischemic heart disease” means a wide range as far as severity can go, while seemingly “healthy” individuals may also have different tolerance of anemia, therefore this is another scenario when “one size does not fit all.”

Indeed, in a prospective human interventional study, it was found that in acute isovolemic anemia with a hemoglobin of 50 g/L in conscious healthy resting humans did not produce hemodynamic instability, but oxygen imbalance was accompanied by a significant drop in SvO_2_ (25). These results were reinforced by a retrospective analysis of a prospective observational study in which ScvO_2_ was found to be a good indicator of transfusion (26). The results of our recent animal study on isovolemic hemodilution gave further evidence that anemia-induced change in oxygen balance can be monitored by ScvO_2_ (27). We found that in anesthetized, ventilated pigs, low hemoglobin levels were well tolerated (59 g/L in this case) without oxygen debt. There was also a strong, negative correlation between VO_2_/DO_2_ and ScvO_2_.

These results suggest that ScvO_2_ may help to identify the point when compensatory mechanisms become exhausted and the relationship between VO_2_/DO_2_ tips the balance, hence in the form ScvO_2_ decision making in transfusion practice is supported by a physiological value rather than just relying on the hemoglobin concentration alone.

Finally, there has also been a paradigm shift in severe bleeding management. There are several new alternatives for improving outcomes of patients in hemorrhagic shock, mainly based on the concept of multimodal hemodynamic support and also on dynamic viscoelastic tests guided individualized coagulation management (28).

It is important to acknowledge the above mentioned “individualized” approaches require well trained, experienced personnel. However, in daily reality patients are often managed by junior staff, especially after hours. Studies have also shown that providing additional hemodynamic information in an everyday setting does not automatically translate into better treatment (29). It is for these situations that simple, “figure driven” protocols have been introduced in the past.

## Summary

There is increasing evidence that interventions based on multimodal monitoring of physiological and biochemical processes in the high-risk patients, let it be in the perioperative or critical care setting, may improve outcome. Advanced knowledge accompanied and reinforced by technical developments of continuous real time monitoring of organ function and molecular mechanisms give firm ground for utilizing them as a “goal directed individualized” approach, which may replace “figure-driven” protocolized care in the future.

## Conflict of Interest Statement

The author declares that the research was conducted in the absence of any commercial or financial relationships that could be construed as a potential conflict of interest.

## References

[1] Pearse RM Moreno RP Bauer P Pelosi P Metnitz P Spies C Mortality after surgery in Europe: a 7 day cohort study. Lancet (2012) 380:1059–65. 10.1016/S0140-6736(12)61148-9 22998715 PMC3493988

[2] Cannesson M Pestel G Ricks C Hoeft AE Perel A. Hemodynamic monitoring and management in patients undergoing high risk surgery: a survey among North American and European anesthesiologists. Crit Care (2011) 15:R197. 10.1186/cc10364 21843353 PMC3387639

[3] Benes J Chytra I Altmann P Hluchy M Kasal E Svitak R Intraoperative fluid optimization using stroke volume variation in high risk surgical patients: results of prospective randomized study. Crit Care (2010) 14:R118. 10.1186/cc9070 20553586 PMC2911766

[4] Salzwedel C Puig J Carstens A Bein B Molnár Z Kiss K Perioperative goal-directed hemodynamic therapy based on radial arterial pulse pressure variation and continuous cardiac index trending reduces postoperative complications after major abdominal surgery: a multi-center, prospective, randomized study. Crit Care (2013) 17:R191. 10.1186/cc12885 24010849 PMC4057030

[5] Pearse RM Harrison DA MacDonald N Gillies MA Blunt M Ackland G Effect of a perioperative, cardiac output-guided hemodynamic therapy algorithm on outcomes following major gastrointestinal surgery a randomized clinical trial and systematic review. JAMA (2014) 311:2181–90. 10.1001/jama.2014.5305 24842135

[6] Pestana D Espinosa E Eden A Najera D Collar L Aldecoa C Perioperative goal-directed hemodynamic optimization using noninvasive cardiac output monitoring in major abdominal surgery: a prospective, randomized, multicenter, pragmatic trial: POEMAS Study. Anesth Analg (2014) 119:579–87. 10.1213/ANE.0000000000000295 25010820

[7] Ackland GL Iqbal S Paredes LG Toner A Lyness C Jenkins N Individualised oxygen delivery targeted haemodynamic therapy in high-risk surgical patients: a multicentre, randomised, double-blind, controlled, mechanistic trial. Lancet Respir Med (2015) 3:33–41. 10.1016/S2213-2600(14)70205-X 25523407

[8] Vallet B Robin E Lebuffe G Venous oxygen saturation as a physio-logic transfusion trigger. Crit Care (2010) 14:213 10.1186/cc8854 20236457 PMC2887106

[9] Mythen MG Webb AR. Intraoperative gut mucosal hypoperfusion is associated with increased post-operative complications and cost. Intensive Care Med (1994) 20:99–104. 10.1007/BF01707662 8201106

[10] Holte K Sharrock NE Kehlet H Pathophysiology and clinical implications of perioperative fluid excess. Br J Anaesth (2002) 89:622–32 10.1093/bja/aef220 12393365

[11] Marik PE Baram M Vahid B. Does central venous pressure predict fluid responsiveness? A systematic review of the literature and the tale of seven mares. Chest (2008) 134:172–8. 10.1378/chest.07-2331 18628220

[12] Osman D Ridel C Ray P Monnet X Anguel N Richard C Cardiac filling pressures are not appropriate to predict hemodynamic response to volume challenge. Crit Care Med (2007) 35:64–8. 10.1097/01.CCM.0000249851.94101.4F 17080001

[13] Molnár Z Vincent JL Still a (valuable) place for the pulmonary artery catheter. Int J Cardiol (2014) 173:131–2 10.1016/j.ijcard.2014.03.040 24681015

[14] Boyd JH Walley KR. The role of echocardiography in hemodynamic monitoring. Curr Opin Crit Care (2009) 15:239–43. 10.1097/MCC.0b013e32832b1fd0 19346938

[15] Németh M Tánczos K Demeter G Érces D Kaszaki J Mikor A Central venous oxygen saturation and carbon dioxide gap as resuscitation targets in a hemorrhagic shock. Acta Anaesth Scand (2014) 58:611–9. 10.1111/aas.12312 24641618

[16] Tánczos K Németh M Molnár Z The hemodynamic puzzle: solving the impossible? In: Vincent JL, editor. Annual Update in Emergency and Critical Care Medicine. Switzerland: Springer International Publishing and BioMed Central (2014). p. 355–65.

[17] De Backer D Heenen S Piagnerelli M Koch M Vincent JL. Pulse pressure variations to predict fluid responsiveness: influence of tidal volume. Intensive Care Med (2005) 31:517–23. 10.1007/s00134-005-2586-4 15754196

[18] Cecconi M Aya HD Central venous pressure cannot predict fluid-responsiveness. Evid Based Med (2014) 19:63 10.1136/eb-2013-101496 24132054

[19] Michard F Boussat S Chemla D Anguel N Mercat A Lecarpentier Y Relation between respiratory changes in arterial pulse pressure and fluid responsiveness in septic patients with acute circulatory failure. Am J Respir Crit Care Med (2000) 162:134–8. 10.1164/ajrccm.162.1.9903035 10903232

[20] Reinhart K Kuhn HJ Hartog C Bredle DL. Continuous central venous and pulmonary artery oxygen saturation monitoring in the critically ill. Intensive Care Med (2004) 30:1572–8. 10.1007/s00134-004-2337-y 15197435

[21] Molnár Z Umgelter A Toth I Livingsone D Weyland A Sakka SG Continuous monitoring of ScvO_2_ by a new fibre-optic technology compared with blood gas oximetry in critically ill patients: a multicentre study. Intensive Care Med (2007) 33:1767–70. 10.1007/s00134-007-0743-7 17576533

[22] Pearse RM Dawson D Fawcett J Rhodes A Grounds RM Bennett ED. Changes in central venous saturation after major surgery, and association with outcome. Crit Care (2005) 9:R694–9. 10.1186/cc3108 16356220 PMC1414025

[23] Collaborative Study Group on Perioperative ScvO_2_ Monitoring. Multicentre study on peri- and postoperative central venous oxygen saturation in high-risk surgical patients. Crit Care (2006) 10:R158. 10.1186/cc5094 17101038 PMC1794462

[24] Westbrook A Pettilä V Nichol A Bailey MJ Syres G Murray L Transfusion practice and guidelines in Australian and New Zealand intensive care units. Intensive Care Med (2010) 36:1138–46 10.1007/s00134-010-1867-8 20440603

[25] Weisskopff RB Viele MK Feiner JB Kelley S Lieberman J Noorani M Human cardiovascular and metabolic response to severe, isovolaemic anaemia. JAMA (1988) 279:217–21. 10.1001/jama.279.3.217 9438742

[26] Adamczyk S Robin E Barreau O Fleyfel M Tavernier B Lebuffe G Contribution of central venous oxygen saturation in postoperative blood transfusion decision. Ann Fr Anesth Reanim (2009) 28:522–30. 10.1016/j.annfar.2009.03.013 19467825

[27] Kocsi S Demeter G Fogas J Érces D Kaszaki J Molnár Z. Central venous oxygen saturation is a good indicator of altered oxygen balance in isovolemic anemia. Acta Anaesthesiol Scand (2012) 56:291–7. 10.1111/j.1399-6576.2011.02622.x 22260228

[28] Tánczos K Németh M Molnár Z What’s new in hemorrhagic shock? Intensive Care Med (2015). 10.1007/s00134-015-3658-8 25608924

[29] Takala J Ruokonen E Tenhunen JJ Parviainen I Jakob SM. Early non-invasive cardiac output monitoring in hemodynamically unstable intensive care patients: a multi-center randomized controlled trial. Crit Care (2011) 15:R148. 10.1186/cc10273 21676229 PMC3219022

